# Mycobacterial antigens in pleural fluid mononuclear cells to diagnose pleural tuberculosis in HIV co-infected patients

**DOI:** 10.1186/s12879-020-05165-6

**Published:** 2020-07-01

**Authors:** Tehmina Mustafa, Ida Wergeland, Kamaldeen Baba, Sharad Pathak, Anwar A. Hoosen, Anne Margarita Dyrhol-Riise

**Affiliations:** 1 Centre for International Health, Department of Global Public Health and Primary Care, University of Bergen, P.O. box 7804, N-5020 Bergen, Norway; 2 Department of Thoracic Medicine, Haukeland University Hospital, Bergen, Norway; 3 Department of Internal Medicine, Haukeland University Hospital, Bergen, Norway; 4 Department of Microbiological Pathology, Medunsa Campus, University of Limpopo, Mankweng, South Africa; 5 Department of Pathology and Laboratory Medicine, King Abdullah bin Abdulaziz University Hospital, Princess Noura bint Abdulrahman University, Riyadh, Saudi Arabia; 6 Department of Respiratory Medicine, Oslo University Hospital, Rikshospitalet, Oslo, Norway; 7 Pathcare - Vermaak & Partners Pathologists and Department of Medical Microbiology, University of Pretoria, Pretoria, South Africa; 8 Department of Infectious Diseases, Oslo University Hospital, Oslo, Norway; 9 Institute of Clinical Medicine, University of Oslo, Oslo, Norway; 10 Department of Clinical Science, University of Bergen, Bergen, Norway

**Keywords:** Mycobacterial antigens, LAM, MPT64, Pleural fluid mononuclear cells, Diagnosis, Pleural tuberculosis, HIV-TB coinfection

## Abstract

**Background:**

Extra pulmonary manifestation of tuberculosis (TB) accounts for approximately one-half of TB cases in HIV-infected individuals with pleural TB as the second most common location. Even though mycobacteria are cleared, mycobacterial antigens may persist in infected tissues, causing sustained inflammation and chronicity of the disease. The aim of this study was to explore various mycobacterial antigens in pleural effusions, the impact of HIV infection and CD4+ T-cell depletion on the presence of antigens, and the diagnostic potential of antigens for improved and rapid diagnosis of pleural TB.

**Methods:**

Pleural fluid specimens were collected from patients presenting with clinically suspected pleural TB, and processed routinely for culture, cytology, and adenosine deaminase activity analysis. HIV status and CD4+ T-cell counts were recorded. Pleural fluid mononuclear cells (PFMC) were isolated, and cell smears were stained with acid-fast staining and immunocytochemistry for various mycobacterial antigens. Real-time and nested-PCR were performed. Patients were categorized as pleural TB or non-TB cases using a composite reference standard. Performance of the mycobacterial antigens as diagnostic test was assessed.

**Results:**

A total of 41 patients were enrolled, of which 32 were classified as pleural TB and 9 as non-TB. Thirteen patients had culture confirmed pleural TB, 26 (81%) were HIV-TB co-infected, and 64% had < 100 CD4+ T-cells/microL. Both secreted and cell-wall mycobacterial antigens were detected in PFMC. Lipoarabinomannan (LAM) was the most frequently detected antigen. There was no direct correlation between positive culture and antigens. Cases with low CD4+ T-cell counts had higher bacterial and antigen burden. By combining detection of secreted antigen or LAM, the sensitivity and specificity to diagnose pleural TB was 56 and 78%, respectively, as compared to 41 and 100% for culture, 53 and 89% for nested PCR, and 6 and 100% for real-time PCR.

**Conclusion:**

Mycobacterial antigens were detectable in PFMC from tuberculous pleural effusions, even in cases where viable mycobacteria or bacterial DNA were not always detected. Thus, a combination of secreted antigen and LAM detection by immunocytochemistry may be a complement to acid-fast staining and contribute to rapid and accurate diagnosis of pleural TB.

## Background

On a global scale, tuberculosis (TB) is a leading cause of death from a single infectious agent. In 2018, 10 million people were estimated to have developed TB disease, and 1.2 million died because of TB. TB remains the number one cause of death in people living with HIV [1]. In South Africa, 60% of adult TB cases are HIV positive [1]. Extrapulmonary TB accounts for approximately one-fifth of TB cases in immune-competent individuals and up to one-half in HIV-infected individuals [2, 3]. In high TB endemic settings, TB of the pleura occurs in up to 30% of TB patients, the second most common site of extrapulmonary involvement following lymph node TB [4]. In the HIV-infected population, pleural TB is the most common cause of a lymphocytic pleural effusion [4–6].

The natural history of TB pleural effusion in HIV-negative individuals is a slow resolution without treatment, although, up to 65% will progress to active TB within 5 years [7, 8]. However, with HIV-coinfection, progression to active disease is more frequent and occurs faster. TB pleural effusions, most probably occur as a result of pleural infection directly from associated parenchymal lesions [9–11]. Immunocompetent individuals are able to mount a strong immune response, and are thus able to control mycobacterial multiplication [12], resulting in paucibacillary forms of TB disease [13]. Paradoxically, the immune response of the host is also responsible for the pathology and chronicity of the disease. It is believed that, even though mycobacteria are cleared, mycobacterial antigens persist in the infected tissue and could be responsible for persistent inflammation and chronicity of disease [14–17].

The pathogenesis of pleural TB in HIV co-infected TB cases is not fully understood. Little is known about the impact of HIV and depletion of CD4+ T-cells on the pathogenesis of pleural TB, or the identity of the mycobacterial antigens persisting in TB pleural effusions.

Studies have shown that people with TB and HIV co-infection with low CD4+ T-cell counts have detectable Lipoarabinomannan (LAM) in urine [18]. This implies that HIV-induced CD4+ T-cell depletion affects the ability of the host to control mycobacterial replication, thereby, leading to higher bacillary load and disseminated disease with higher amounts of mycobacterial antigens at the disease site.

We have previously shown that the expression of mycobacterial antigens in pulmonary TB is distinct from that of extrapulmonary TB [17]. Of note, we have specifically demonstrated that the secreted mycobacterial antigen MPT64 is preferentially expressed in and co-localized with TB lesions, leading to the hypothesis that this protein is accumulated in inflammatory cells and could be responsible for persistence of disease [17, 19–25]. The detection of secreted mycobacterial antigen MPT64 by immunochemistry from the aspirates, effusions or biopsies has been evaluated as a useful and better diagnostic test for various forms of extrapulmonary TB [17, 19–25].

The aim of this study was to explore the expression of various mycobacterial antigens in the cells from pleural effusions, the impact of HIV infection and CD4 + T-cell depletion on the accumulation of mycobacterial antigens, and the diagnostic potential of mycobacterial antigens from pleural effusion for improved and rapid diagnosis of pleural TB.

## Methods

### Study participants

Pleural fluid analyses were performed on stored samples from a previous published study [26]. Briefly, patients presenting with pleural effusion and clinical symptoms of pleural TB admitted to the Dr. George Mukhari Hospital, Ga-Rankuwa, Pretoria, South Africa during the period 2004–2005 were recruited into the study. Patients with recent TB therapy (< 1 year) or treated with corticosteroids, immunosuppressive or antiretroviral therapy were not included. HIV testing was done routinely in pleural effusion patients with suspected TB. The demographic and clinical data, HIV status and CD4+ T-cell count were recorded for each patient.

### Specimen collection and processing

Thoracocentesis was performed according to clinical practice at the hospital. The pleural fluid specimens were processed according to standard laboratory routines for acid fast bacilli (AFB) staining of smears and culture (BacT-alert, Org anon, Teknika). The pleural fluid was also sent for cytological examination and Adenosine deaminase activity (ADA) analysis using a commercial colorimetric assay kit (Diazyme General Atomics, CA) with a cut-off value for positive test of 30 U/L [27, 28]. At the time of inclusion pleural fluid mononuclear cells (PFMCs) were isolated from approximately 200 ml pleural fluid by density gradient centrifugation (Ficoll histopaque 1077, Sigma). Cells were washed once with isotonic NaCl, and twice with RPMI with 10% fetal calf serum (FCS) (1640, L-glutamine and HEPES supl., Sigma), and resuspended in RPMI media to make a final concentration of 1 × 10^6^ cells/ml. Cells were cryopreserved in 10%DMSO/90%FCS. Cryovials were placed in − 80 °C, and then transferred into liquid nitrogen for long term storage. The cells were shipped to Haukeland University Hospital, Bergen, Norway for further analysis. Cryovials were thawed in a 37 °C water bath until the cell suspension was almost melted. The cell suspension was then transferred to 5 mL centrifuge tubes containing RPMI with 10% FCS at 37 °C, centrifuged at 350 g for 5 min, and resuspended in RPMI with 10% FCS. Smears were made for Ziehl Neelsen staining and immunocytochemical staining.

### Immunocytochemical staining

All antibodies used in the study were in-house rabbit polyclonal, except for anti-Bacille Calmette- Guѐrin (BCG), which was obtained from DAKO Immunoglobulins, Copenhagen, Denmark (code B124; lot 063B). Table 1 shows the immunogens used for production of these antibodies, target antigens, how they are named in the manuscript, and dilutions. The specificity of these antisera has been determined by earlier studies [21, 29].

**Table 1:** Primary polyclonal antibodies, immunogens used for the production of antibodies, target antigens, the name used in the study, and dilutions used for immunohistochemistry

Antibody	Immunogen	Target antigens	Study name	Dilution
Anti-MPT64	Antigen extracted and purified from the 5 weeks old culture filtrate of *M. tuberculosis* with minimal lysis*.* The term MPT was introduced by Nagai et al. for the designation of proteins purified from *M.tuberculosis.* The number is based on their relative mobility in 7.7% polyacrylamide gel electrophoresis gels at a running pH of 9.5 [29].	MPT64 (Rv1980c). Secreted antigen specific for *M.tuberculosis* complex organisms. Absent from most of atypical mycobacteria.	secreted antigen	1:50
Anti-Antigen 60	Cell wall antigens of *M.tuberculosis*. The number is based on their relative mobility as mentioned above [29]	Major *M.tuberculosis* cell wall antigens.	Cell wall antigens	1:100
Anti- Lipoarabinomannan (LAM)	LAM	LAM, the primary component of *M.tuberculosis* cell wall.	LAM	1:100
Anti-Bacille Calmette- Gue’rin (BCG)	Bacterial sonicates of BCG containing both secreted and cell wall antigens.	Most of the secreted and cell wall antigens of the *M. tuberculosis* based on the similarity between *M.tuberculosis* and BCG.	Heterogeneous antigens	1:2000

Immunocytochemistry was performed by using the DakoCytomation kit (EnVision + System-HRP; DakoCytomation Denmark A/S, Glostrup, Denmark). The PFMC smears were passed through graded alcohol and treated with 3% bovine serum albumin for 5 min. Primary antibodies were added, and the smears incubated for 1 h 15 min. Optimal dilutions were determined by titration. The smears were incubated with anti-rabbit dextran polymer conjugated to horseradish peroxidase for 45 min. The endogenous peroxidase activity was inhibited by incubating the smears with hydrogen peroxide for 8 min. Antigens were visualized by incubation with 3-amino-9-ethylcarbazol and hydrogen peroxide containing substrate for 15 min. The smears were counter-stained with hematoxylin. All incubations were carried out at room temperature and the smears were washed thoroughly between incubations.

Negative and positive controls were included in all experiments. One smear where the primary antibody was substituted with antibody diluent, and one smear from a patient with non-tuberculous pleural effusion were used as negative controls. One smear from a culture positive patient with abundant acid-fast bacilli on Ziehl-Neelsen staining was used as positive control.

### Evaluation of immunostaining

The stained slides were evaluated at 10x magnification using a light microscope and positive signals were further assessed at 40x magnification. Cells with intracellular granular staining were counted at 10x magnification. The whole smear was examined, and the number of fields counted. The antigen load was quantified by calculating the number of stained cells per 100 fields. For diagnostic purpose, a case with any positive signal was labelled as positive.

### Nested polymerase chain reaction (PCR) for IS6110

DNA extraction was performed by adding 400-1000 μL of the PFMC suspension to a cryotube containing 250 μl of acid-washed micro-glass beads and ribolysing the tubes for 45 s. A 123- base pair fragment from IS6110 was amplified using the following primers 5′ CCTGCGAGCGTAGGCGTCGG 3′ and 5′ CTCGTCCAGCGCCGCTTCGG 3′. The product was subjected to a second round of PCR amplification using the primers 5′ TTCGGACCACCAGCACCTAA 3′ and 5′ TCGGTGACAAAGGCCACGTA 3′ to amplify a 92-base pair fragment. The PCR reaction mixture consisted of 5 μL DNA, 25 μL Taq master mix (Qiagen), 0.25 μl of each 100 μM primer stock solution, and distilled water to make a final volume of 50 μl. For the second round of PCR, 1 μL of the first PCR product was used as template. The reaction cycle for the first PCR was- 94 °C for 1 min, 68 °C for 1 min, 72 °C for 20 s for 45 cycles, and for the nested-PCR - 94 °C for 1 min, 58 °C for 1 min, and 72 °C for 20 s for 35 cycles. Both PCR had an initial heat activation step of 95 °C for 15 min and a final extension of 72 °C for 10 min. The amplified products were analyzed in a 3% agarose gel stained with ethidium bromide. Mycobacterial DNA isolated from bacterial cultures was used as positive control and a reaction tube where test template was substituted with distilled water as negative control in each PCR run. A negative control tube containing RPMI, going through all steps including the DNA extraction protocol were also included.

**Fig. 1:**
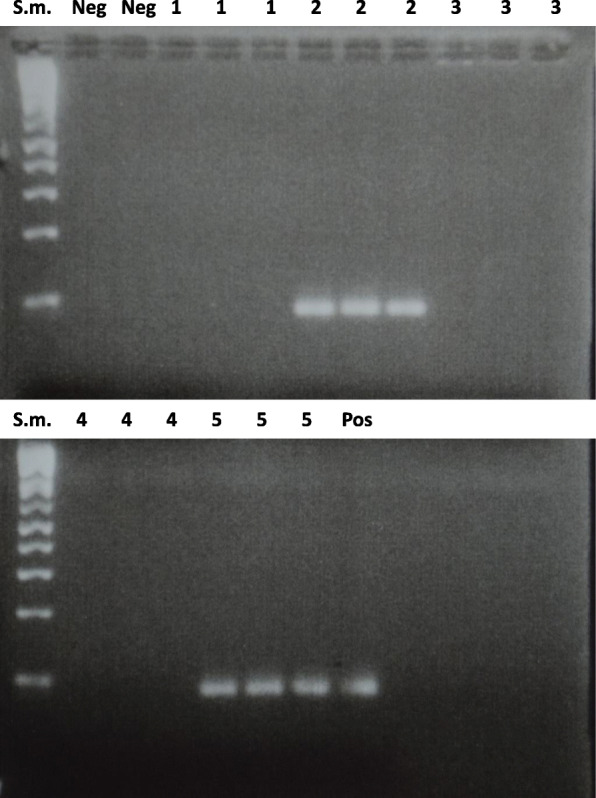
Gel electrophoresis of the nested-PCR showing PCR amplification of a 92-bp fragment of IS6110. Results of five samples are shown, with triplicates from each sample. Only sample 2 and 5 are positive. Lane s.m.; size marker. Neg; negative control. Pos; positive control

Mycobacterial DNA load was estimated based on the number of nested-PCR positive triplicates from each sample. Low and high load was defined when PCR was positive in one of the three, and three of three samples, respectively. For diagnostic purposes, any positive in the triplicate was labelled as positive. The gel electrophoresis image of the nested-PCR results for some of the samples is shown in Fig. 1.

### Real-time PCR

A real-time PCR assay targeting a 103-base pair long segment of the mycobacterial heat shock protein 65 gene *GroEL2* was performed as previously published by our group [30]. The target segment was amplified using the forward primer MycoFP1 (5′ -CGAGGCGATGGACAAGGT-3′), the reverse primer TB12 (5′ -CTTGTCGAACCGCATACCCT-3′), and the fluorescent-tagged Taq Man MGB probe MycoPr1 (5′ -VIC-AACGAGGGCGTCATCACCGTCG-MGB-3′). The PCR reactions were set up in a total volume of 20 μl consisting of 10 μL of 2 × TaqMan Universal PCR MasterMix (Applied Biosystems), 5 μL DNA extract from clinical specimen and a final concentration of each primer and probe of 0.9 μM and 0.25 μM, respectively. Amplification was done with a 7500 Fast-Real-Time System (Applied Biosystems) using the following parameters: 2 min at 50 °C to activate AMPErase UNG, 10 min at 95 °C to activate AmpliTaq Gold, followed by 45 thermal cycles of 95 °C for 15 s and 60 °C for 1 min. Each clinical specimen was run in technical duplicates or triplicates. To further ensure the reliability of results, each run included both negative controls (5 μL of sterile water) as well as several positive controls (known dilutions of genomic DNA from Bacille Calmette- Gue’rin, Mycobacterium spp. ATCC 19015D). Standard quantification curves were generated based on the known concentrations of the positive controls using the Sequence Detection System software v1.3 (Applied Biosystems). As per recommendations of the manufacturer (Applied Biosystems), real-time PCR data were analyzed using a threshold set at 10 SDs above background signal level and only threshold cycle values below 40 were noted as positive.

### Patient categories

The patients were categorized by using a composite reference standard by combining the clinical and laboratory diagnostic criteria. Pleural TB was defined based on either mycobacterial confirmation by AFB microscopy and/or culture positive pleural fluid, or in the absence of bacteriological confirmation by clinical criteria based on ADA ≥30 U/L from the pleural fluid and/or strong clinical evidence consistent with active TB followed by a clinical decision to treat with anti-tuberculosis therapy [26]. A case was categorized as non-TB when diagnosed as malignancy or another non-TB condition.

### Statistical analysis

Data was analyzed using Statistical Package for the Social Sciences for Windows version 25.0. The data was not normally distributed, thus non-parametric tests were used for two group comparisons. Wilcoxon signed-ranked test was used for matched analysis, and Mann–Whitney test was used for two independent group comparisons. Chi-square test was used for comparison of ratios between two groups. Spearman’s rank correlation was used to determine the relationship between variables. A *p*-value of < 0.05 was considered significant. Cross-tabulation was used to calculate the sensitivities and specificities of diagnostic tests.

### Ethical considerations

The study was evaluated and approved by the Research, Ethics and Publications Committee at the Dr. George Mukhari Hospital, University of Limpopo, South Africa (2003) and the Regional Committee for Ethics in Medical Research in Bergen, Norway (2003, REK Vest nr.185.02). All the patients received written and verbal information of the study and all gave informed written consent.

## Results

### Patient characteristics

Table 2 shows the demographic and laboratory characteristics of the 41 patients enrolled in the study. Case based information is provided in Additional file 1. Thirty-two (78%), were classified as pleural TB cases based on the composite reference standard, and nine as non-TB cases. Thirteen patients had culture confirmed pleural TB, 26 (81%) were HIV pleural TB co-infected, and 64% of these had < 100 CD4+ T cells/microL. The median ADA level was 62 U/L. Among non-TB cases, 5 (56%) were HIV positive and median ADA levels were 15 U/L. Seven of the study participants had a history of previous TB with anti-tuberculous treatment finished more than 1 year before study inclusion.

**Table 2:** Demographic and laboratory characteristics of patients

Characteristics ***n*** = 41	Number
Gender	
Females	16 (39%)
Males	25 (61%)
Age, median (range)	36 (19–69)
**Pleural TB cases**	32 (78%)
Diagnosis based on	
Culture	13 (41%)
AFB microscopy +, ADA > 30 and clinical evidence	1 (3%)
ADA > 30 and clinical evidence	17 (53%)
Clinical evidence only^b^	1 (3%)
HIV positive	26 (81%)
CD4+ T cell count HIV+, (n) median (range)^a^	(22) 80 (7–328)
CD4+ T cell count > 100 cells/microL	8 (36%)
CD4+ T cell count < 100 cells/microL	14 (64%)
ADA levels U/L (n) median (range)^a^	(29) 62 (4–200)
**Non-TB cases**	9 (22%)
Malignancy	3 (33%)
Another non-TB condition ^c^	6 (67%)
HIV positive	5 (56%)
CD4+ T cell count HIV+, (n) median (range)^a^	(4) 119 (49–375)
CD4+ T cell count > 100 cells/microL	3 (75%)
CD4+ T cell count < 100 cells/microL	1 (25%)
ADA levels U/L (n) median (range)a	(9) 15 (1–29)

*n* number, ^a^results were not available for some patients

^b^ADA level just below cutoff

^c^Pleural effusion of other known etiology such as congestive cardiac failure or para-pneumonic effusion

### Culture, PCR and mycobacterial antigens in the pleural fluid mononuclear cells according to the TB and HIV status

Among the HIV pleural TB co-infected, the proportion of culture positive cases was significantly higher as compared to the HIV-negative pleural TB cases (50% versus 0%, respectively), while the proportion of AFB microscopy, nested-PCR and real-time PCR was not different (Table 3). Among the non-TB cases, there was no difference between HIV positive and HIV negative cases, except for a higher number of positive for a combination of culture or nested-PCR or any antigen in the HIV positive group (Table 3).

**Table 3:** Results of various procedures on pleural fluid mononuclear cells

	TB cases (***n*** = 32)		Non-TB (***n*** = 9)	
	HIV+ (***n*** = 26)	HIV- (***n*** = 6)	HIV+ (***n*** = 5)	HIV- (***n*** = 4)
AFB microscopy+ n (%)	3 (12%)	1 (17%)	0 (0%)	0 (0%)
Culture+ n (%)	13 (50%)	0 (0%)*	0 (0%)	0 (0%)
Nested PCR+ n (%)	15 (58%)	2 (33%)	1 (20%)	0 (0%)
Real-time PCR+ n (%)	2 (8%)	0 (0%)	0 (0%)	0 (0%)
**Mycobacterial antigens by immunocytochemistry**				
Heterogeneous antigens+ n (%)	11 (42%)	2 (33%)	4 (80%)	0 (0%)
Cell wall antigen+ n (%)	10 (38%)	2 (33%)	1 (20%)	1 (25%)
LAM+ n (%)	13 (50%)	2 (33%)	0 (0%)	0 (0%)
Secreted antigen+ n (%)	9 (35%)	3 (50%)	2 (40%)	0 (0%)
Any antigen+ n (%)	19 (73%)	4 (67%)	4 (80%)	1 (25%)
LAM or secreted antigen+ n (%)	14 (54%)	4 (67%)	2 (40%)	0 (0%)
LAM and secreted antigen+ n (%)	8 (31%)	1 (17%)	0 (0%)	0 (0%)
Heterogeneous antigens stained cells/100 fields median (range)	0 (0–348)	0 (0–66)	8 (0–31)	0 (0–0)
Cell wall antigen stained cells/100 fields median (range)	0 (0–130)	0 (0–19)	0 (0–1)	0 (0–1)
LAM, stained cells/100 fields median (range)	0.5 (0–33)	0 (0–12)	0 (0–0)	0 (0–0)
Secreted antigen stained cells/100 fields median (range)	0 (0–72)	2 (0–12)	0 (0–5)	0 (0–0)
**Combinations of antigens and other diagnostic tests**				
Culture and/or n-PCR and/or any antigen+ n (%)	23 (89%)	5 (83%)	5 (100%)*	1 (25%)
Culture and/or n-PCR and/or LAM n (%)	21 (81%)	4 (67%)	1 (20%)	0 (0%)

*significant difference between HIV positive and HIV negative by Pearson chi-square, *p* value < 0.05

**Fig. 2:**
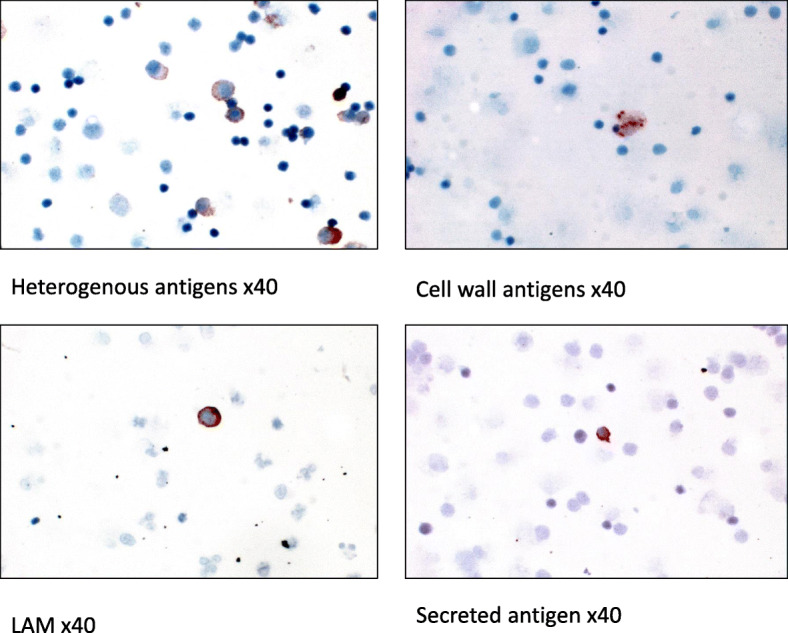
Cell smears from the mononuclear cells from tuberculous pleural effusions showing the staining pattern of mycobacterial antigens as detected by immunocytochemical staining. Staining pattern is intracellular granular in the cytoplasm of pleural fluid mononuclear cells

**Fig. 3:**
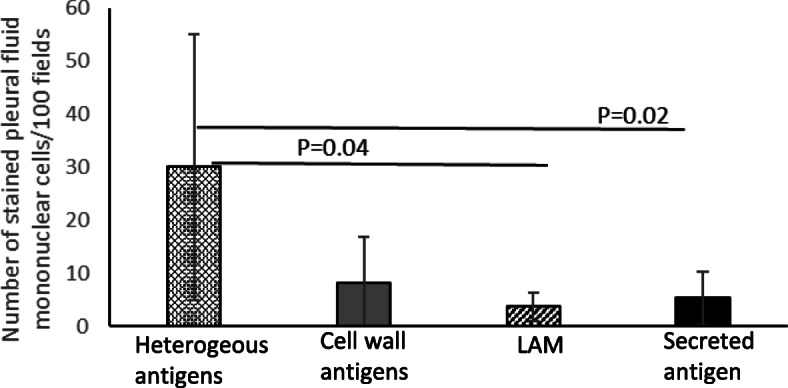
The mycobacterial antigen load among pleural TB cases (*n* = 32) measured by the number of stained pleural fluid mononuclear cells per 100 fields. Wilcoxon Signed Ranks Test for paired samples was used for comparisons

Mycobacterial antigens were observed as intracellular granular staining in the cytoplasm of pleural fluid mononuclear cells (Fig. 2). There was no difference in the proportion of mycobacterial antigen positive cases or load of any antigens between HIV positive and HIV negative pleural TB cases (Table 3). The load of individual antigens varied among pleural TB cases. Heterogeneous antigens were the most abundant (Fig. 3) and were higher as compared to LAM (*p* = 0.04) and secreted antigen (*p* = 0.02) (Fig. 3). The levels of antigens had a significant positive correlation with each other, except between heterogeneous mycobacterial antigens and cell wall antigen (Table 4). Interestingly, culture had a tendency towards negative correlation with all antigens, but the association was not statistically significant (Table 4).

**Table 4:** Relationship between culture, nested-PCR, bacterial DNA load, CD4+ T cell count and levels of mycobacterial antigens in the tuberculous pleural fluid mononuclear cells based on Spearman’s rank correlation. The values shown are the correlation coefficients

	Culture	DNA load	Heterogeneous antigens	Cell wall antigen	LAM	Secreted antigen
DNA load	0.467^b^					
Heterogeneous antigens	0.198	0.155				
Cell wall antigen	−0.067	0.270	0.259			
LAM	0.217	0.079	0.442^a^	0.370^a^		
Secreted antigen	0.032	0.088	0.625^b^	0.663^b^	0.439^a^	
N-PCR	−0.394^a^	− 0.938^b^	− 0.236	−0.117	− 0.110	−0.055
CD4+ T cell count	−0.507^b^	−0.341	− 0.184	−0.272	− 0.050	−0.122

^a^Correlation is significant at the 0.05 level (2-tailed), ^b^Correlation is significant at the 0.01 level (2-tailed). *N-PCR* Nested PCR

DNA load is measured based on the number of positives among triplets from each case

**Fig. 4:**
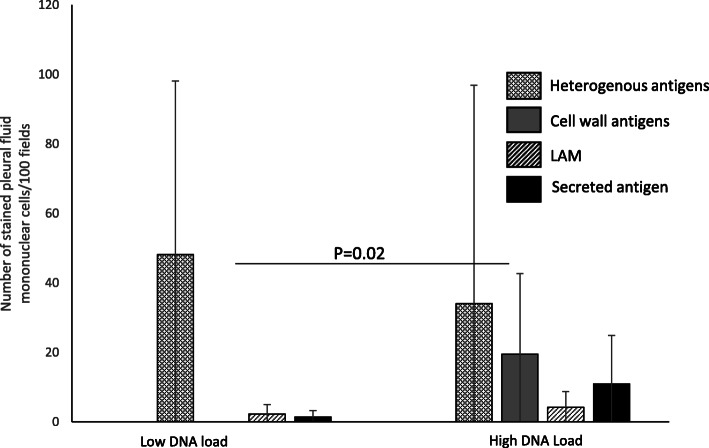
Difference in the mycobacterial antigen load among cases with higher and lower DNA load (*n* = 18). Mycobacterial DNA load is categorized based on the number of positives with nested-PCR among the triplets from each specimen. Mann-Whitney test was used for comparisons

Association of mycobacterial DNA load with culture, AFB microscopy and the mycobacterial antigens was also studied. Cases with higher DNA load had higher number of culture positive (*p* = 0.06), microscopy positive (*p* = 0.07) and cell wall antigen positive (*p* = 0.007) results as compared to those with lower DNA load (Table 5). Figure 4 shows the load of various antigens among cases with higher and lower DNA load. The load of cell wall antigen was higher in the cases with higher DNA load (*p* = 0.02), while there was no difference in the load of any of the other mycobacterial antigens studied (Fig. 4). Additional file 1 provides case-based information.

**Table 5:** Relationship of CD4+ T cell count in HIV-pleural TB co-infected (*n* = 22,CD4+ T cell count not available for four patients), and the mycobacterial DNA load in all nested-PCR positive patients (*n* = 18) with AFB microscopy, culture and the mycobacterial antigens by Pearson chi-square test

	CD4 > 100	CD4 < 100	*p*-value	DNA low^a^	DNA high^a^	*p*-value
culture+	2	9	0.07	2	8	0.06
culture-	6	5		5	3	
Microscopy +	0	3	0.15	0	4	0.07
Microscopy -	8	11		7	7	
Cell wall antigen+	0	9	0.003	0	7	0.007
Cell wall antigen-	8	5		7	4	
Heterogeneous antigens+	2	8	0.14	4	5	0.62
Heterogeneous antigens-	6	6		3	6	
LAM+	3	8	0.37	4	5	0.62
LAM-	3	6		3	6	
secreted antigen+	1	8	0.04	2	5	0.47
secreted antigen-	7	6		5	6	
N-PCR+	3	9	0.22			
N-PCR-	5	5				
RT-PCR+	0	2	0.26	0	2	0.23
RT-PCR-	8	12		7	9	

*N-PCR* Nested PCR, *RT-PCR* Real time PCR

^a^DNA load is based on the number of positives among triplets from each case, low = 1/3, high = 3/3 positive

### Association of CD4+ T-cell count with the mycobacterial antigens and bacterial load in pleural TB patients

**Fig. 5:**
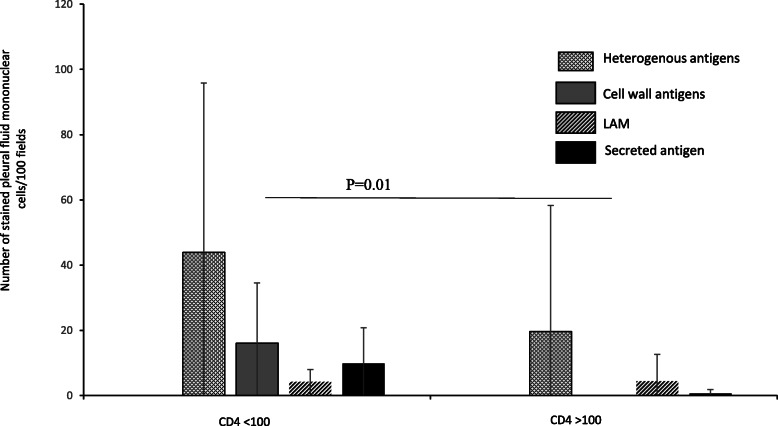
Difference in the mycobacterial antigen load among pleural TB cases according to the CD4+ T cell count (*n* = 27, CD4+ T cell count not available for five cases). Antigen load is measured by the number of stained pleural fluid mononuclear cells per100 fields. Mann-Whitney test was used for comparisons

Pleural TB cases with CD4+ T-cell counts < 100 had higher number of culture positive cases as compared to cases with CD4+ T-cell counts > 100 (*p* = 0.07) (Table 5), indicating higher viable bacterial load. There was a significant negative correlation between the CD4+ T-cell counts and culture (*p* = 0.006) (Table 4). The number of cases positive for cell wall antigen (*p* = 0.003) and secreted antigen (*p* = 0.04) were also higher in those with CD4+ T cell count < 100 (Table 5). The load of individual antigens was higher among cases with CD4+ T-cell count < 100 as compared to those with CD4+ T-cell count > 100. The only statistically significant difference was seen for cell wall antigen (*p* = 0.01), where this antigen was not detected in cases with higher CD4+ T-cell counts (Fig. 5). There was a trend towards negative correlation between CD4+ T-cell counts and the mycobacterial antigens, though without statistical significance (Table 4). Additional file 1 provides case-based information.

### Performance of various mycobacterial antigens as diagnostic test

Using the composite reference standard, sensitivity and specificity of the individual antigens to diagnose pleural TB was between 38 and 47% and 56–100% respectively (Table 6). LAM was the most frequently detected antigen (47%) and was not detected in any of the non-TB cases. When using culture or the combination of culture and nested-PCR as reference standard, the sensitivity of the individual antigens was, in general, higher. Except for heterogenous antigens, the specificity, was lower as compared to the composite reference standard.

**Table 6:** Diagnostic validity of different tests performed on pleural fluid samples by using three reference standards

	CRS		Culture		Culture & N-PCR	
	SN	SP	SN	SP	SN	SP
Culture	41	100				
Nested-PCR	53	89	77	71		
Real-time PCR	6	100	15	100	12	100
AFB Microscopy	12	100	23	96	23	100
Mycobacterial antigens						
Cell wall antigen	38	78	31	64	41	71
LAM	47	100	54	71	53	75
Heterogeneous antigens	41	56	54	64	47	62
Secreted antigen	38	78	39	68	35	67
Secreted antigen or LAM	56	78	54	54	59	58
Any 4 antigens	72	44	69	32	76	37
Culture, nested-PCR or any antigen	88	33				
Culture, nested-PCR or LAM	78	89				

*CRS* Composite reference standard, *SN* Sensitivity, *SP* Specificity

Among rapid tests, the sensitivity of antigen detection was better than the AFB microscopy and real-time PCR, and very close to the nested-PCR. The sensitivity of culture was 41%. By using a composite antigen detection approach based on the detection of any of the four mycobacterial antigens, the sensitivity improved to 72%, which was better than all the other tests, although the specificity was reduced to 44% (Table 6).

Using culture as a reference standard, the sensitivity of the individual antigens was better than AFB microscopy and real-time PCR, but lower than the nested-PCR. By using a combination of culture and nested-PCR as reference standard, the sensitivity of antigen detection was much better than the AFB microscopy and real-time PCR (Table 6).

### Head-to-head comparison of various diagnostic methods

As the different diagnostic tests were positive in different cases, a head-to-head comparison of all tests was also performed to show that combination of various tests gives a better diagnostic yield as compared to a single test (Table 7). Among the 23 nested-PCR negative cases, 10 more cases were positive with combination of secreted antigen and LAM, and 15 more cases with combination of all antigens. A combination of culture, nested-PCR, and mycobacterial antigens was positive in 28 (88%) of pleural TB cases, indicating added value of combining various diagnostic tests.

**Table 7:** Head-to-head comparison of different diagnostic tests among all cases

	Culture +	Culture -	Microscopy+	Microscopy-	N-PCR+	N-PCR-	RT-PCR+	RT-PCR-	Cell wall antigen+	Cell wall antigen-	Hetero- geneous antigens+	Hetero-geneous antigens-	LAM+	LAM-	Secreted antigen+	Secreted antigen-
	*n* = 13	*n* = 28	*n* = 4	*n* = 37	*n* = 18	*n* = 23	*n* = 2	*n* = 39	*n* = 14	*n* = 27	*n* = 17	*n* = 24	*n* = 15	*n* = 26	*n* = 14	*n* = 27
Microscopy+	3	1														
Microscopy-	10	27														
N-PCR +	10	8	4	14												
N-PCR -	3	20	0	23												
RT-PCR+	2	0	2	0	2	0										
RT-PCR-	11	28	2	37	16	23										
Cell wall antigen+	4	10	3	11	7	7	2	12								
Cell wall antigen-	9	18	1	26	11	16	0	27								
Heterogenous antigens+	7	10	3	14	9	8	2	15	7	10						
Heterogenous antigens-	6	18	1	23	9	15	0	24	7	17						
LAM+	7	8	2	13	9	6	2	13	8	7	9	6				
LAM-	6	20	2	24	9	17	0	26	6	20	8	18				
Secreted antigen+	5	9	3	11	7	7	2	12	9	5	10	4	9	5		
Secreted antigen-	8	19	1	26	11	16	0	27	5	22	7	20	6	21		
LAM or secreted antigen+	7	13	3	17	10	10	2	18	10	10	12	8	15	5	14	6
LAM or secreted antigen-	6	15	1	20	8	13	0	21	4	17	5	16	0	21	0	21
Any antigen+	9	19	4	24	13	15	2	26	14	14	17	11	15	13	14	14
Any antigen-	4	9	0	13	5	8	0	13	0	13	0	13	0	13	0	13

*N-PCR* Nested PCR, *RT-PCR* Real time PCR

## Discussion

In this study, we have shown that PFMC from tuberculous pleural effusions detain secreted and cell wall associated mycobacterial antigens. HIV coinfection had an impact on the bacterial burden, shown by a significantly higher number of culture positive effusion in the HIV pleural TB co-infected group. In addition, in the HIV pleural TB co-infected group, the cases with low CD4+ T cell count had a higher number of culture positives and higher loads of cell wall antigen and secreted antigen. These findings imply that low CD4+ T-cell counts impair the host ability to clear the infection, resulting in persistence of bacilli and accumulation of mycobacterial antigens. Earlier studies have shown that HIV co-infected pulmonary TB patients with lower CD4+ T-cell count have detectable LAM in the urine, indicating higher burden of LAM with CD4 + T-cell depletion [18]. In our study, the LAM was not significantly higher expressed in the HIV cases with low CD4+ T-cell count, while cell wall antigen and secreted antigen was higher. These differences may be due to the differential accumulation of various mycobacterial antigens at different sites of infection as shown earlier [17], due to the relatively small sample size, or due to different affinities and specificities of the primary antibodies.

Mycobacterial antigens are shown to accumulate in the infected tissues [17, 19–25, 31]. As antigens accumulate as a result of bacterial replication, one would expect a positive correlation between presence of viable bacteria and the antigens. However, a tendency towards negative correlation was seen with cell wall antigens, though the relationship was not statistically significant. This could be due to the persistence of antigens in the infected cells, while bacteria are killed with the advent of an apt immune response, as shown previously in the murine pulmonary TB lesions [31]. These accumulated antigens could play a role in the persistence of inflammation and chronicity of disease. This may explain the low yield of culture in pleural TB, where pathology is sustained by antigens rather than viable bacteria, underlining the importance of measuring antigens in culture negative cases when TB diagnosis is uncertain.

The secreted antigen MPT64 constitutes a small fraction of the secreted antigens in in vitro culture filtrates [29]. On the other hand, in vivo it is detectable in quantities comparable to other major cell-wall associated antigens. This is most probably due to accumulation of this antigen in the infected cells. Our previous study on pleural biopsies from the same hospital have shown that this antigen is detectable in 80% of the pleural TB biopsies, while only 20% of these biopsies were positive with culture [19]. In the present study, this antigen was detected in 38% of cases, which is much lower as compared to other studies using the same antibody [20, 23, 25]. This could be due to the isolation and storage of cells before staining in this study. The freeze-thaw process could have resulted in the degradation of antigen. In other studies, the smears were prepared from fresh specimens which were fixed immediately.

LAM is a promising mycobacterial antigen detection test for pulmonary TB in HIV TB co-infected patients [18]. In our study, LAM was the most abundant antigen in the PFMC and was detectable in 47% of pleural TB cases. By combining detection of secreted antigen or LAM, 56% of cases were detected, which is higher than other tests used in TB diagnostics. The specificity of this combination was 78% compared with 100% for detection of LAM alone. Thus, antigen detection by immunocytochemistry is a robust and rapid test and may assist the clinical decision to start treatment before culture results are available. This would reduce diagnostic delay and curb empirical over-treatment in the absence of bacterial confirmation. Unlike PCR, this method is not sensitive to contamination and can be implemented in a basic pathology lab in high TB endemic settings, without the need for high tech equipment. However, this needs to be evaluated on a larger sample to ensure reproducibility.

One disadvantage of the antigen detection by immunocytochemistry was the number of false positive tests. This could be caused by non-specific binding of the antibodies. However, some results could be true positives, as implied by the higher false positivity in the HIV positive cases. TB is a common co-morbidity in high TB endemic settings, and due to atypical clinical presentation of TB in HIV co-infected patients, some cases could be misdiagnosed in the absence of bacteriological confirmation.

Pleural TB is known to be a paucibacillary disease and this is also confirmed in our study, as only 12% of the cases were positive with direct microscopy, 41% with culture, and only 6% with real-time PCR. The low sensitivity of real-time PCR is similar to the results of Xpert MTB/RIF assay on pleural effusions [20]. WHO has not recommended the Xpert MTB/RIF assay for diagnosis of pleural effusions due to low sensitivity [32]. Nested-PCR was positive in 53% cases. However, this PCR is not suitable for routine use in high TB endemic setting due to risk of contamination. Even though there was positive correlation between the cases positive with culture and DNA load, there was a negative correlation between culture positivity and nested-PCR. This finding implies that DNA might persist in the tissues, even if the bacilli are killed as shown previously in murine tissues [33].

## Conclusion

Mycobacterial antigens were detectable in PFMC from tuberculous pleural effusions by immunocytochemistry. There was no direct correlation between antigens and culture, implying that antigens persist in the infected tissues despite clearance of bacilli. More cases can be diagnosed by combination of antigen detection and culture. A combination of LAM and secreted antigen could diagnose more cases of pleural TB as compared to all the routine tests. This immunocytochemistry-based method is rapid and robust, and unlike PCR, it is not sensitive to contamination. Hence, it can contribute to the timely management of pleural TB cases in high TB and HIV endemic settings. Further studies are required to test the reproducibility of the test.

## Supplementary information

**Additional file 1.**

## Data Availability

The datasets used and/or analyzed during the current study are provided as an additional file.

## Abbreviations

ADA: Adenosine deaminase activity
AFB: Acid fast bacilli
BCG: Bacille Calmette- Guѐrin
FCS: Fetal calf serum
LAM: Lipoarabinomannan
PCR: Polymerase Chain Reaction
PFMC: Pleural fluid mononuclear cells
TB: Tuberculosis
